# Novel peptides for deciphering structural and signalling functions of E-cadherin in mouse embryonic stem cells

**DOI:** 10.1038/srep41827

**Published:** 2017-02-07

**Authors:** Joe M. Segal, Christopher M. Ward

**Affiliations:** 1 Stem Cell Research Group, The University of Manchester, AV Hill Building, Oxford Road, Manchester, M13 9PT, United Kingdom

## Abstract

We have previously shown that E-cadherin regulates the naive pluripotent state of mouse embryonic stem cells (mESCs) by enabling LIF-dependent STAT3 phosphorylation, with E-cadherin null mESCs exhibiting over 3000 gene transcript alterations and a switch to Activin/Nodal-dependent pluripotency. However, elucidation of the exact mechanisms associated with E-cadherin function in mESCs is compounded by the difficulty in delineating the structural and signalling functions of this protein. Here we show that mESCs treated with the E-cadherin neutralising antibody DECMA-1 or the E-cadherin binding peptide H-SWELYYPLRANL-NH_2_ (Epep) exhibit discrete profiles for pluripotent transcripts and NANOG protein expression, demonstrating that the type of E-cadherin inhibitor employed dictates the cellular phenotype of mESCs. Alanine scanning mutation of Epep revealed residues critical for *Tbx3, Klf4* and *Esrrb* transcript repression, cell-cell contact abrogation, cell survival in suspension, STAT3 phosphorylation and water solubility. STAT3 phosphorylation was found to be independent of loss of cell-cell contact and Activin/Nodal-dependent pluripotency and a peptide is described that enhances STAT3 phosphorylation and *Nanog* transcript and protein expression in mESCs. These peptides represent a useful resource for deciphering the structural and signalling functions of E-cadherin and demonstrate that complete absence of E-cadherin protein is likely required for hierarchical signalling pathway alterations in mESCs.

E-cadherin is a single-pass transmembrane glycoprotein which functions to facilitate calcium-dependent homotypic cell adhesion in epithelial tissues. E-cadherin maintains cytoskeletal dynamics through linkage of the cytoplasmic domain to the actin cytoskeleton via β-catenin^1^. E-cadherin is critical for mammalian development as mice lacking the protein fail to develop beyond the blastocyst stage^2^, reflecting loss of epithelial integrity in both the trophectoderm and inner cell mass^2^,^3^. The cytoplasmic region of E-cadherin binds to β-catenin, allowing interaction with the actin cytoskeleton via intermediate proteins, such as α-catenin^4^,^5^. In addition, p120^ctn^ binds to the juxta-membrane region of the E-cadherin cytoplasmic domain and contributes to stabilisation of the cadherin-catenin complex by preventing clathrin-mediated endocytosis^6^. E-cadherin-mediated cell-cell contact can respond to “outside-in” and “inside-out” cues that reflect a range of cellular functions^6^, demonstrating the complex and critical role of this protein in epithelial tissue homeostasis. Loss of cell surface E-cadherin is a also defining characteristic of epithelial-mesenchymal transition (EMT), which is required for ingression of epiblast cells within the primitive streak during early embryonic development^1^,^7^ and is associated with tumour cell metastasis^8^,^9^.

Mouse embryonic stem cells (mESCs) are isolated from the inner cell mass (ICM) of blastocysts and can maintain pluripotency *in vitro* by culture in the presence of serum (containing bone morphogenetic proteins (BMPs)) and the cytokine Leukaemia Inhibitory Factor (LIF) by activation of STAT3 and SMAD1/5/8 signalling^10^,^11^. We have previously shown that E-cadherin null (Ecad^−/−^) mESCs exhibit a significantly altered transcriptome compared to wild type (wt) ESCs, including downregulation of transcripts associated with the naïve pluripotency regulatory network^12^. However, elucidation of the exact mechanisms associated with E-cadherin function in mESCs is compounded by the difficulty in delineating the structural and signalling functions of this protein. For example, abrogation of E-cadherin in mESCs leads to a more polarized actin cytoskeleton organisation^13^ which is associated with Ecad−/− mESCs switching from LIF/BMP- to Activin/Nodal-dependent pluripotency^14^. However, the exact mechanism associated with this switch is not clear: it may reflect altered E-cadherin signalling via STAT3 phosphorylation^15^ which directly influences the pluripotent phenotype, or it may be an indirect effect due to the altered actin cytoskeleton activating/inhibiting unknown proteins/pathways. Therefore, at present it remains unknown whether the transcriptional and post-translational modifications associated with loss of E-cadherin are a result of direct or indirect (or both) regulation via E-cadherin.

E-cadherin provides an attractive target to manipulate ESCs in culture since cell signalling mediated through this protein has significant effects on both ESC pluripotent states and survival. We have previously shown that abrogation of E-cadherin-mediated cellular aggregation allows culture of mESCs in shake flask bioreactors whilst retaining pluripotency, either through gene knockout or an inhibitory antibody DECMA-1^16^. However, utilisation of E-cadherin neutralising Abs for ESC culture is expensive and more cost-effective E-cadherin inhibitors are required before this technique becomes common practise. Devemy and Blaschuk^17^ have previously reported the generation of a dual E/N-cadherin binding peptide, referred to here as ‘Epep’, which induces reversible loss of cell-cell contact via *trans-*homophilic cadherin binding inhibition. The Epep sequence SWELYYPLRANL was identified by phage display library generation and the Tryptophan (TRP) residue at position 2 is believed to mimic the binding activity of a conserved TRP residue at amino acid position 2 within the ectodomain of the mature E-cadherin protein^17^. Epep has been shown to inhibit E-cadherin mediated cell-cell contact in MCF-7 and mESC cell lines, and was able to abrogate phosphorylation of STAT3 in the latter^15^,^17^. In addition, we have shown that Ecad−/− mESCs cultured in clonal grade medium (LIF/BMP) re-establish cell-cell contact and STAT3 phosphorylation via N-cadherin^15^ and treatment with Epep abolished cell-cell contact and STAT3 phosphorylation.

In this study we have used mutational analysis of Epep to determine residues responsible for solubility, cell-cell contact inhibition, cell survival in suspension, STAT3 phosphorylation inhibition and transcriptional regulation of the naïve-associated genes *Nanog, Klf4, Tbx3, Esrrb, Nr0b1* and *Nr5a2*. We show that Epep exhibits distinct functional properties compared to the E-cadherin neutralising antibody DECMA-1, demonstrating that the type of E-cadherin inhibitor employed dictates the transcriptional phenotype observed in mESCs. Furthermore, this suggests that different regions of E-cadherin regulate different aspects of mESC pluripotency. A minimal sequence required for Epep function is shown and alanine scanning mutational analysis of Epep enabled identification of residues responsible for solubility, inhibition of cell-cell contact and STAT3 phosphorylation, and regulation of *Nanog, Klf4, Esrrb* and *Tbx3* transcripts. Overall, our data demonstrates that the structural and signalling functions of E-cadherin can be demarcated using a range of peptides based on the Epep sequence, which will allow further analysis of the function of this protein in mESC pluripotency to be investigated.

## Results

### Abrogation of E-cadherin mediated cell-cell contacts in mESCs using Epep leads to repression of pluripotency associated transcripts and STAT3 phosphorylation

**Figure 1:**
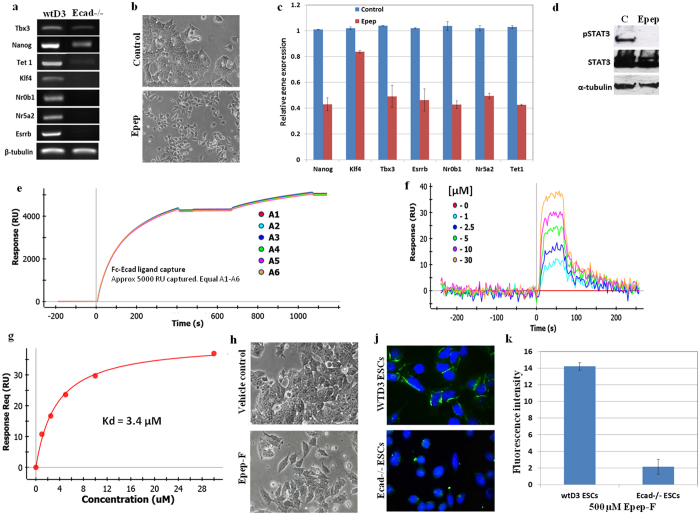
Abrogation of E-cadherin in mESCs leads to repression of transcripts associated with naïve pluripotency. (**a**) RT-PCR analysis of *Tbx3, Nanog, Tet1, Klf4, Nr0b1, Nr5a2,* and *Esrrb* transcripts in wtD3 and E-cadherin−/− mESCs. (**b**) Phase contrast microscopy images of wtD3 mESCs treated with control vehicle or the E-cadherin binding peptide Epep. (**c**) qPCR analysis of *Nanog, Klf4, Tbx3, Esrrb, Nr0b1, Nr5a2* and *Tet1* transcript expression in mESCs treated with vehicle control or Epep. (**d**) Western blot analysis of phosphorylated STAT3 (pSTAT3), total STAT3 and α-tubulin proteins in wtD3 mESCs treated with control vehicle (C) or Epep. (**e**) Biacore Surface Plasmon Resonance (SPR) analysis was performed using human recombinant E-cadherin-Fc chimaera protein bound to an HTE chip by high capacity His-tagged protein capture. Capture of the recombinant E-cadherin-Fc chimaera protein on 6 independent HTE chips for high capacity His-tagged protein capture (A1-A6) over time is shown. (**f**) Biacore SPR equilibrium binding analysis using affinity capture of Epep at 0, 1, 2.5, 5, 10 and 30 μM. (**g**) Concentration curve of Epep binding to recombinant E-cadherin-Fc chimaera protein at various concentrations to enable K_D_ calculation. (**h**) Phase contrast image showing that fluorescent tagged Epep (Epep-F) is able to inhibit cell-cell contact in wtD3 mESCs. (**j**) Immunofluorescence microscopy analysis of Epep-F binding (green) to Ecad^−/−^ and wtD3 mESCs following incubation at 500 μm for 15 minutes at 37 °C. Nuclei are stained with DAPI (Blue). (**k**) Quantification of Epep-F binding to wtD3 and Ecad^−/−^ mESCs using ImageJ analysis. Data shows the mean fluorescence intensity and standard deviation. Epep - SWELYYPLRANL.

RT-PCR analysis in wild-type (wt)D3 and Ecad^−/−^ ESCs demonstrated absence or decreased expression of *Tbx3, Nanog, Tet1, Klf4, Nr0b1, Nr5a2* and *Esrrb* transcripts in the latter (Fig. 1a). wtD3 ESCs treated with Epep exhibited loss of cell-cell contact within 24 h (Fig. 1b) and statistically significant decreased expression of *Nanog, Klf4, Tbx3, Esrrb, Nr0b1, Nr5a2* and *Tet1* transcripts compared to control treated cells (Fig. 1c; all p < 0.05). Furthermore, Epep-treated wtD3 ESCs exhibited significantly decreased phosphorylation of STAT3 compared to control treated cells (Fig. 1d). Therefore, treatment of wtD3 ESCs with Epep results in a similar phenotype to that observed in Ecad−/− ESCs for *Nanog, Klf4, Tbx3, Esrrb, Nr0b1, Nr5a2* and *Tet1* transcript expression and STAT3 phosphorylation. Biacore Surface Plasmon Resonance (SPR) analysis was performed using mouse E-cadherin-Fc recombinant chimaera protein capture (Fig. 1e) and equilibrium analysis using affinity capture showed the K_D_ for Epep to be 3.4 μM (Fig. 1f and g). To further confirm the specific binding of Epep to E-cadherin a fluorescein-5(6)-isothiocyanate label was conjugated to the NH_2_-terminus of the peptide (Epep-F) separated by a Nova linker molecule to prevent the fluorescent label interfering with the peptide binding function. Treatment of wtD3 ESCs with Epep-F resulted in loss of cell-cell contact (Fig. 1h), preferential binding to wtD3 ESCs compared to Ecad−/− cells, as evidenced by fluorescence microscopy analysis (Fig. 1j), and statistically significant increased binding to wtD3 over Ecad−/− ESCs, as determined by fluorescence flow cytometry analysis (Fig. 1k; p < 0.0001 unpaired *t*-test).

### Epep and DECMA-1 induced inhibition of E-cadherin mediated cell-cell contacts in mESCs result in differential NANOG expression, gene transcript alterations and cellular viability

**Figure 2:**
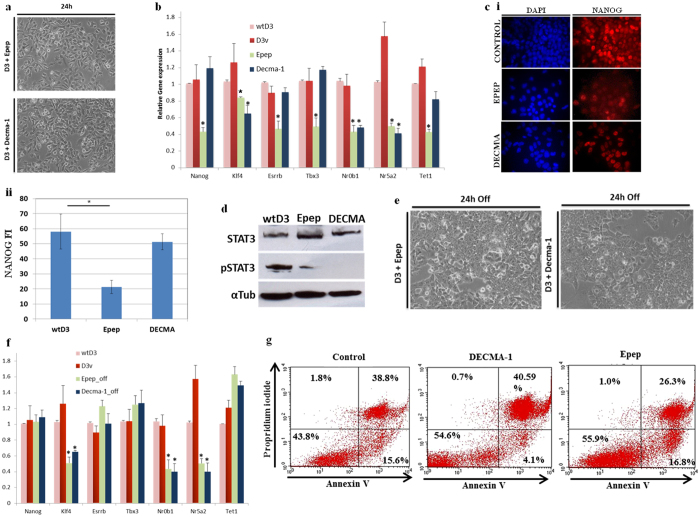
Inhibition of E-cadherin in mESCs using Epep or DECMA-1 results in differential gene transcript expression. (**a**) Phase contrast images of mESCs treated with Epep or DECMA-1 neutralising antibody for 24 h. (**b**) qPCR analysis of *Nanog, Klf4, Esrrb, Tbx3, Nr0b1, Nr5a2* and *Tet1* transcript expression in mESCs treated with vehicle control (D3v), Epep or DECMA-1. (**c**) (i) Immunofluorescence microscopy analysis of NANOG protein expression in wtD3 mESCs treated with vehicle control (Control), Epep or DECMA-1 (DECMA). Nuclei are stained with DAPI (Blue). (ii) Quantification of NANOG protein expression in the images shown in (i) using ImageJ analysis. Data shows the mean fluorescence intensity and standard deviation. (**d**) Western blot analysis of phosphorylated STAT3 (pSTAT3), total STAT3 and α-tubulin proteins in wtD3 mESCs treated with control vehicle (wtD3), Epep or DECMA-1 (DECMA). (**e**) Phase contrast images of wtD3 mESCs exhibiting restoration of cell-cell contact 24 h post-removal of Epep or DECMA-1 treatment. (**f**) qPCR analysis of *Nanog, Klf4, Esrrb, Tbx3, Nr0b1, Nr5a2* and *Tet1* transcript expression in wtD3 mESCs 24 h post-removal of Epep (Epep_off) or DECMA-1 (Decma-1_off) treatment. (**g**) Apoptosis analysis using Annexin V and propridium iodide fluorescence flow cytometry in wtD3 mESCs treated with vehicle control (Control), DECMA-1 or Epep. The lower left box of the flow cytometry plots shows the viable population, the lower right box the early apoptotic cells and the top right box the late apoptotic or necrotic cells. Epep - SWELYYPLRANL.

We next compared the transcriptional alterations associated with Epep treatment of mESCs to that of the E-cadherin neutralising antibody DECMA-1, which bind the EC1 and EC5 extracellular domains of E-cadherin respectively. Both Epep- and DECMA-1-treatment of wtD3 ESCs resulted in loss of cell-cell contact after 24 h (Fig. 2a) and qPCR analysis demonstrated statistically significant downregulation of *Klf4, Nr0b1* and *Nr5a2* transcripts in both treatments, whilst *Nanog, Esrrb, Tbx3* and *Tet1* transcripts were decreased only upon Epep treatment (Fig. 2b). Epep treated mESCs exhibited decreased NANOG protein expression (Fig. 2c; 96 h shown) whilst both Epep and DECMA-1 treatment decreased STAT3 phosphorylation within 24 h (Fig. 2d). Removal of the E-cadherin antagonists from wtD3 ESCs shown in Fig. 2b led to restoration of cell-cell contact (Fig. 2e), however, *Klf4, Nr0b1* and *Nr5a2* transcripts remained significantly decreased at 24 h following removal of both Epep and DECMA-1 (Fig. 2f). To determine the signalling pathways associated with transcript expression following treatment of wtD3 ESCs with Epep or DECMA-1, we cultured mESCs with the E-cadherin antagonists in the presence of small molecule inhibitors of the TGFβ, MEK and AKT pathways (Figure S1a). The Akt pathway was implicated in the inhibition of *Esrrb, Tbx3* and *Nr5a2* transcripts and the TGFβ pathway was associated with repression of *Klf4* and *Nr5a2* transcripts in Epep-treated cells (Figure S1c). In DECMA-1-treated mESCs, TGFβ pathway inhibition had no effect on any transcript expression levels, whereas, Akt inhibition led to decreased *Tbx3* transcripts and MEK/ERK inhibition resulted in decreased *Esrrb* transcripts (Figure S1c). This data shows that different E-cadherin inhibitors exhibit different signalling pathway effects on pluripotency transcripts in mESCs.

To determine the effect of Epep and DECMA-1 on the viability of mESCs, we assessed apoptosis in the cell populations following treatment with the E-cadherin antagonists for 24 h using Annexin V and propridium iodide fluorescence flow cytometry analysis (Fig. 2g). The lower left box of the flow cytometry plots shows the viable population, the lower right box the early apoptotic cells and the top right box the late apoptotic or necrotic cells. DECMA-1 treated mESCs exhibited decreased early apoptotic cells and increased viable cells compared to control treated mESCs (Fig. 2g; DECMA-1) whilst Epep-treated cells exhibited no statistically significant changes in early apoptotic cell numbers but showed decreased late apoptotic/necrotic cells compared to control treated mESCs. Therefore, exogenous abrogation of E-cadherin using DECMA-1 or Epep increases cell viability compared to control-treated cells, although the mechanisms for this increased survival appear to be distinct.

### Truncated and W2R mutant peptides exhibit differential activity compared to Epep

**Figure 3:**
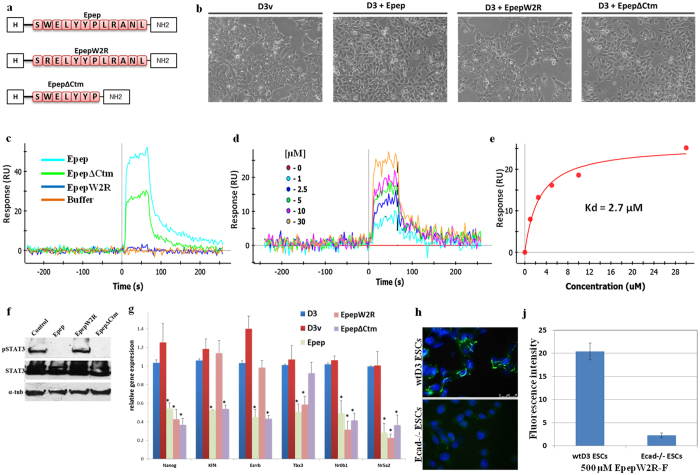
Analysis of specific residues required for Epep induced loss of cell-cell contact and the minimum residues required for this function. (**a**) Diagrammatic representation of Epep, EpepW2R and Epep∆ctm peptides used in the analysis. (**b**) Phase contrast images of mESCs treated with Epep, EpepW2R or Epep∆ctm for 24 h. (**c**) Biacore SPR equilibrium binding analysis of Epep, EpepW2R or Epep∆ctm at 30 μM. (**d**) Biacore SPR equilibrium binding analysis of Epep∆ctm at 0, 1, 2.5, 5, 10 and 30 μM and (**e**) binding curve for the calculation of peptide K_D_. (**f**) Western blot analysis of phosphorylated STAT3 (pSTAT3), total STAT3 and α-tubulin (α-tub) proteins in wtD3 mESCs treated with control vehicle (wtD3), Epep, EpepW2R or Epep∆ctm. (**g**) qPCR analysis of *Nanog, Klf4, Esrrb, Tbx3, Nr0b1* and *Nr5a2* transcript expression in mESCs treated with vehicle control (D3v), Epep, EpepW2R or Epep∆ctm at 10 μM. (**h**) Immunofluorescence microscopy analysis of EpepW2R-F binding (green) to Ecad^−/−^ and wtD3 mESCs following incubation at 500 μm for 15 minutes at 37 °C. Nuclei are stained with DAPI (Blue). (**j**) Quantification of EpepW2R-F binding to wtD3 and Ecad^−/−^ mESCs using ImageJ analysis. Data shows the mean fluorescence intensity and standard deviation. Epep – SWELYYPLRANL; EpepW2R – SRELYYPLRANL; Epep∆ctm – SWELYYP.

Within the Epep sequence a tryptophan residue at position 2 is believed to mimic the action of *trans* homotypic binding by inserting into a hydrophobic pocket within the E-cadherin EC1 domain^17^. A peptide with the Trp-2 residue replaced with arginine in the Epep sequence (EpepW2R; Fig. 3a) was synthesised to assess the influence of the Trp residue on peptide binding to E-cadherin and activity. In addition, a truncated version of Epep (EpepΔCtm) was assessed to determine the minimal number of residues required for peptide activity (Fig. 3a). Peptides smaller than EpepΔCtm were insoluble in water and were not tested further. Treatment of mESCs with EpepW2R did not induce loss of cell-cell contact after 24 h (Fig. 3b; D3 + EpepW2R) whereas EpepΔCtm-treated cells exhibited loss of cell-cell contact similar to that observed for Epep (Fig. 3b; D3 + EpepΔCtm). This shows that the tryptophan residue at position 2 in Epep is required for loss of cell-cell contact, as described by Devemy and Blaschuk^17^ for MCF7 cells, and that the five terminal amino acid residues are redundant for abrogation of E-cadherin mediated cell-cell contact. The binding affinity of peptides EpepW2R and EpepΔCtm were assessed using Biacore SPR analysis (Fig. 3c). Sensorgram analysis of Epep, EpepW2R and EpepΔCtm peptides (Fig. 3d) showed increased binding to Fc-E-cadherin of EpepΔCtm over EpepW2R with the steady state response curve (Fig. 3e) predicting a K_D_ of 2.7 μM for EpepΔCtm. EpepW2R did not produce a strong enough response to perform equilibrium analysis, although later analysis of a EpepW2A variant exhibited a K_D_ of 55 μM. Whilst the K_D_ for EpepΔCtm is lower than Epep (K_D_ = 3.4 μM) the response is proportional to the molecular weight therefore smaller molecules give lower molar response. Therefore, the binding efficiency of Epep and EpepΔCtm was not considered to be significantly different.

**Figure 4:**
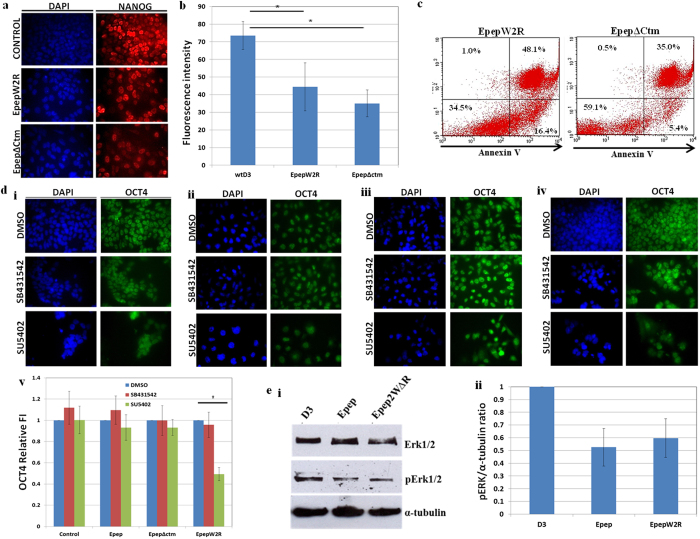
Epep treatment of mESCs does not induce maintenance of pluripotency via the Activin/Nodal signalling pathways. (**a**) Immunofluorescence microscopy analysis of NANOG protein expression in wtD3 mESCs treated with vehicle control (Control), EpepW2R or Epep∆ctm. Nuclei are stained with DAPI (Blue). (**b**) Quantification of NANOG protein expression in the images shown in (**a**) using ImageJ analysis. Data shows the mean fluorescence intensity and standard deviation. (**c**) Apoptosis analysis using Annexin V and propridium iodide fluorescence flow cytometry in wtD3 mESCs treated with EpepW2R or Epep∆ctm. The lower left box of the flow cytometry plots shows the viable population, the lower right box the early apoptotic cells and the top right box the late apoptotic or necrotic cells. (**d**) Immunofluorescence microscopy analysis of OCT4 protein expression in wtD3 mESCs treated with the Activin/Nodal inhibitor SB431542 or the FGFR1 inhibitor SU5402 and (i) vehicle control, (ii) Epep, (iii) EpepΔCtm and (iv) EpepW2R. (v) Relative fluorescence intensity of OCT4 protein expression in (i)-(iv) assessed using ImageJ analysis. (**e**) (i) Western blot analysis of phosphorylated ERK1/2 (pERK1/2), total ERK1/2 and α-tubulin proteins in wtD3 mESCs treated with control vehicle (D3), Epep or EpepW2R. (ii) Graph showing the ratio of pERK1/2 and α-tubulin protein levels from the western blot in **e**(i) using ImageJ densitometry analysis. Epep – SWELYYPLRANL; EpepW2R – SRELYYPLRANL; Epep∆ctm – SWELYYP.

EpepΔCtm-treated mESCs exhibited significantly decreased phosphorylation of STAT3 (Fig. 3f) whilst this was unaffected in EpepW2R-treated cells. qPCR analysis of *Nanog, Klf4, Tbx3, Esrrb, Nr0b1 and Nr5a2* transcript expression was assessed in wtD3 ESCs treated with Epep, EpepW2R or EpepΔCtm (Fig. 3g). EpepΔCtm-treated mESCs exhibited similar alterations in transcript expression compared to Epep except for *Tbx3*, which was unchanged, suggesting that the 5 terminal residues of Epep are required for repression of *Tbx3* transcripts. Surprisingly, EpepW2R-treated mESCs exhibited a statistically significant decrease in *Nanog, Tbx3, Nr0b1 and Nr5a2* transcript expression, whilst *Klf4* and *Esrrb* transcripts were unchanged. EpepW2R binds specifically to E-cadherin as evidenced by immunofluorescence microscopy analysis of binding of a fluorescent-tagged EpepW2R peptide to wtD3 and Ecad−/− ESCs (Fig. 3h and j; p < 0.0001 unpaired *t*-test). Analysis of NANOG protein expression in mESCs treated with EpepW2R or EpepΔCtm revealed decreased levels of protein with both peptide treatments after 4 days (Fig. 4a and b). Annexin V/PI apoptosis analysis of EpepW2R-treated mESCs revealed a significant decrease in viable cell number and an increase in late apoptotic/necrotic cells (Fig. 4c; EpepW2R) compared to untreated cells (Fig. 2g). EpepΔCtm-treated mESCs exhibited a significant decrease in early apoptotic cell numbers and increased viable cells (Fig. 4c; EpepΔCtm) compared to both untreated and Epep-treated mESCs (Fig. 2j), although late apoptotic/necrotic cell numbers were increased compared to Epep.

We have previously shown that Ecad−/− mESCs maintain pluripotency via the Activin/Nodal pathways and self-renewal via the FGFR1 pathway^14^. To determine whether peptide-treated mESCs exhibited a switch from LIF/BMP to Activin/Nodal and FGFR1 signalling we exposed the peptide-treated mESCs to small molecule inhibitors^14^,^15^ and assessed expression of OCT4 protein after 5 days. mESCs treated with the Activin/Nodal inhibitor SB431542 or the FGFR1 inhibitor SU5402 maintained expression of OCT4 protein (Fig. 4d(i) and (v)). Similarly, wtESCs treated with Epep (Fig. 4d(ii)) or EpepΔCtm (Fig. 4d(iii)) maintained expression of OCT4 protein in the presence of SB431542 or SU5402 (Fig. 4d(v)). EpepW2R-treated wtD3 mESCs (Fig. 4d(iv) and (v)) exhibited similar results to control cells in the presence of the Activin/Nodal inhibitor SB431542 but showed increased cell death and statistically significant loss of OCT protein expression when exposed to the FGFR1 inhibitor SU5402. To determine whether EpepW2R exhibited an effect on downstream signalling targets of FGFR1, we assessed phosphorylation of ERK1/2 proteins in mESCs treated with Epep and EpepW2R (Fig. 4e). Epep- and EpepW2R-treated cells exhibited decreased pERK1/2 compared to untreated mESCs. Therefore, Epep, EpepΔCtm and EpepW2R do not induce Activin/Nodal-dependent pluripotency in mESCs, however, EpepW2R appears to induce the FGFR1 pathway for maintenance of OCT4 protein expression and cell survival.

### The proline-7 residue is required for water solubility of Epep

**Figure 5:**
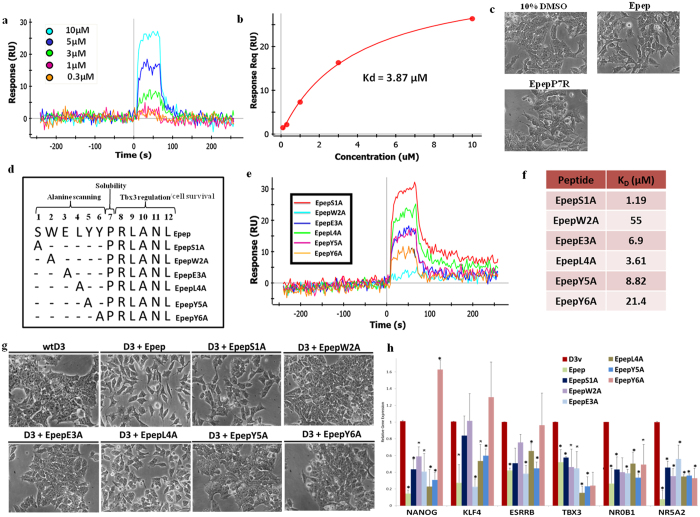
Alanine scanning mutational analysis of Epep reveals discrete residues associated with transcript repression and solubility. (**a**) Biacore SPR equilibrium binding analysis using affinity capture of EpepP7R at 0.3, 1, 3, 5, 10 μM. (**b**) Concentration curve of EpepP7R binding to recombinant E-cadherin-Fc chimaera protein at various concentrations to enable K_D_ calculation. (**c**) Phase contrast images of wtD3 mESCs treated with vehicle control (10% v/v DMSO in H_2_0), Epep and EpepP7R. (**d**) The alanine scanning mutations synthesised based on the sequence of Epep. (**e**) Biacore SPR equilibrium binding analysis using affinity capture of EpepS1A, EpepW2A, EpepE3A, EpepL4A, EpepY5A and EpepY6A. (**f**) K_D_ values for EpepS1A, EpepW2A, EpepE3A, EpepL4A, EpepY5A and EpepY6A calculated using Biacore SPR equilibrium affinity capture analysis. (**g**) Phase contrast images of wtD3 mESCs treated with vehicle control (wtD3), Epep, EpepS1A, EpepW2A, EpepE3A, EpepL4A, EpepY5A and EpepY6A at 10 μM. (**h**) qPCR analysis of *Nanog, Klf4, Tbx3, Esrrb, Nr0b1* and *Nr5a2* transcript expression in mESCs treated with vehicle control (D3v), Epep, EpepS1A, EpepW2A, EpepE3A, EpepL4A, EpepY5A and EpepY6A at 10 μM. Epep - SWELYYPLRANL; EpepS1A – AWELYYPLRANL; EpepW2A – SAELYYPLRANL; EpepE3A – SWALYYPLRANL; EpepL4A – SWEAYYPLRANL; EpepY5A – SWELAYPLRANL; EpepY6A – SWELYAPLRANL; EpepP7R – SWELYYRLRANL.

Devemy and Blaschuk^17^ described a conserved proline residue at position 7 in all seven acid eluted peptide sequences and seven out of nine sequences of EDTA elutions in the phage display screening analysis, suggesting this residue may have an important functional role. Analysis of a peptide with the Pro-7 residue replaced with Arg, EpepP7R, revealed that this peptide was insoluble at 100 mM in water and required 10%v/v DMSO/H_2_O for complete solubilization. The binding affinity of EpepP7R was assessed using Biacore SPR analysis (Fig. 5a) revealing a K_D_ of 3.87 μM (Fig. 5b), which was slightly higher than that of Epep dissolved in 10%v/v DMSO/H_2_O (1.15 μM; data not shown). Abrogation of E-cadherin mediated cell-cell contact was observed with EpepP7R (Fig. 5c and Figure S1b), although cell dissociation was reduced compared to Epep. Therefore, the Pro-7 residue within Epep is required for water solubility of the peptide.

### Alanine scanning mutational analysis of Epep reveals specific residues required for pluripotent transcript repression, STAT3 phosphorylation and loss of cell-cell contact

We next assessed the function of the first 6 amino acid residues within Epep using alanine scanning mutational analysis^18^ (Fig. 5d). The relative binding affinity of the alanine scanned variants was determined (Fig. 5e) and dissociation constants calculated by equilibrium binding (Fig. 5f). The alanine mutation at serine 1 (EpepS1A) had the least detrimental effect on E-cadherin binding affinity (Fig. 5f) and this peptide exhibited similar cell-cell contact abrogation activity compared to Epep (Fig. 5g and Figure S1c). EpepL4A exhibited a slightly increased K_D_ of 3.61 μM compared to Epep (K_D_ 3.4 μM; Fig. 5f) and inhibited cell-cell contact in mESCs (Fig. 5g and Figure S1c). Residues 2 (EpepW2A), 3 (EpepE3A), 5 (EpepY5A) and 6 (EpepY6A) exhibited K_D_ of 55, 6.9, 8.82 and 21.4 μM, respectively, and all failed to inhibit cell-cell contact (Fig. 5g and Figure S1c). Therefore, the ability of individual peptides to abrogate cell-cell contact in mESCs appears to be a direct function of their binding affinity for E-cadherin. Fluorescent-tagged EpepS1A, EpepW2A, EpepE3A, EpepL4A, EpepY5A and EpepY6A bind specifically to E-cadherin as evidenced by immunofluorescence microscopy analysis of binding to wtD3 and Ecad−/− ESCs (Figure S2; all p < 0.0001 unpaired *t*-test).

**Figure 6:**
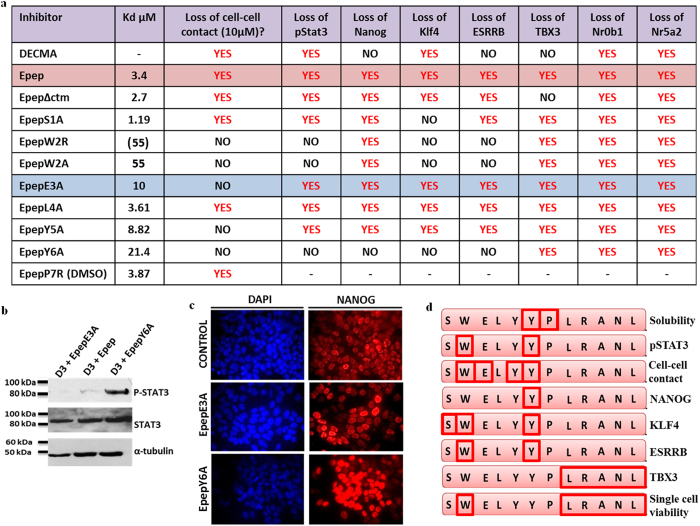
The function of specific residues in Epep related to transcript expression, solubility, cell-cell contact and STAT3 phosphorylation. (**a**) Effect of different peptides and DECMA-1 neutralising antibody on cell-cell contact, STAT3 phosphorylation and transcripts expression in mESCs. (**b**) Western blot analysis of phosphorylated STAT3 (pSTAT3), total STAT3 and α-tubulin proteins in wtD3 mESCs treated with Epep, EpepE3A or EpepY6A. (**c**) Immunofluorescence microscopy analysis of NANOG protein expression in wtD3 mESCs treated with vehicle control (Control), EpepE3A or EpepY6A. Nuclei are stained with DAPI (Blue). (**d**) Summary of the residues within Epep and their function in mESCs ascertained from the mutational peptide analysis. Epep - SWELYYPLRANL; EpepS1A – AWELYYPLRANL; EpepW2R – SRELYYPLRANL; EpepW2A – SAELYYPLRANL; EpepE3A – SWALYYPLRANL; EpepL4A – SWEAYYPLRANL; EpepY5A – SWELAYPLRANL; EpepY6A – SWELYAPLRANL; EpepP7R – SWELYYRLRANL.

qPCR analysis of *Nanog, Klf4, Esrrb, Tbx3, Nr0b1* and *Nr5a2* transcript expression in D3 mESCs after 24 h treatment with the alanine scanned peptide variants showed that all induced down-regulation of *Tbx3, Nr0b1* and *Nr5a2* transcripts (Fig. 5h). Downregulation of *Nanog, Klf4* and *Esrrb* transcripts was found to be dictated by tyrosine residue 6 of Epep, with *Klf4* and *Esrrb* also dependent upon the Trp-2 residue, whilst *Klf4* alone was abrogated by the Ser-1 residue. EpepE3A, EpepL4A and EpepY5A inhibited all gene transcripts assessed, showing that loss of cell-cell contact alone is not responsible for the transcriptional effects of Epep. *Nr0b1* and *Nr5a2* transcripts were downregulated by all of the peptides assessed, suggesting that regulation of these transcripts may be independent of the core pluripotency factor network. All peptides except EpepW2R, EpepW2A and EpepY6A inhibited phosphorylation of STAT3 protein (Fig. 6a). Figure 6a summarises the data obtained from all peptides assessed and DECMA-1 neutralising antibody in mESCs. EpepL4A exhibits a similar function and K_D_ to Epep, suggesting a structural, rather than functional, role for this residue within the peptide. EpepW2R and EpepW2A impart a similar phenotype to mESCs, showing this residue is critical for loss of cell-cell contact and inhibition of STAT3 phosphorylation. EpepE3A is of particular interest as this peptide did not induce loss of cell-cell contact yet inhibited STAT3 phosphorylation (Fig. 6b), exhibited a higher proportion of cells with decreased NANOG protein expression (Fig. 6c) and led to down-regulation of *Nanog, Klf4, Esrrb, Tbx3, Nr0b1* and *Nr5a2* transcripts (Fig. 6a). This suggests that E-cadherin signalling is likely to regulate STAT3 phosphorylation independently of cell-cell contact. EpepY6A is also of interest as mESCs treated with this peptide exhibited elevated levels of Nanog transcripts (Fig. 5h) and protein (Fig. 6c and Figure S1d) and increased pSTAT3 (1.4-fold increase; p < 0.05) compared to control cells, although *Tbx3, Nr0b1* and *Nr5a2* transcripts were all reduced. A summary of the structure-function of Epep is shown in Fig. 6d.

## Discussion

Genetic knockout of the *Cdh1* gene in mESCs results in over 3000 gene transcript alterations associated with a broad range of biological processes^12^. A recent comprehensive proteomics study to map protein interactions involved in cell-cell adhesion revealed a complex E-cadherin interactome network far beyond the well characterized cadherin-catenin complex, with over 400 novel E-cadherin interactions identified^19^. E-cadherin is now considered to be a master regulator of stem cell fate^20^,^21^, a key mediator in the transition between naïve and primed-like pluripotent states^22^ and required for efficient reprogramming of somatic cells to iPSCs^23^. In this study we have described novel peptides based upon the sequence of Epep that can be used to manipulate mESC pluripotency transcripts and phosphorylation of STAT3 protein via both cell-cell contact-dependent and -independent mechanisms. These peptides will provide a valuable resource for deciphering the signalling and structural roles of E-cadherin in regulating pathways in mESCs and other cell types. Further manipulation of the amino acid residues within Epep (e.g. multiple residue substitution) may allow the development of further novel peptides with defined biological function. Whilst we cannot eliminate off-target effects associated with the peptides, all lacked binding to α4β7 control protein, exhibited μM affinity for E-cadherin (both assessed by biacore analysis) and bound wtD3 mESCs but not E-cadherin null mESCs (as assessed by immunofluorescence microscopy). We consider this significant evidence for peptide binding specificity for E-cadherin.

Comparison of the Epep-treated mESC phenotype with that of DECMA-1 treated mESCs revealed significant differences between the two treatments. DECMA-1 treatment of mESCs did not inhibit *Nanog, Esrrb* or *Tbx3* transcripts even though STAT3 phosphorylation was abolished. This demonstrates that the method of E-cadherin inhibition dictates the cellular phenotype observed, which further complicates the elucidation of the function of this protein in mESC pluripotency. This result suggests that specific regions of E-cadherin exhibit discrete functions in mESCs. We have previously reported that phosphorylation of AKT, which has been shown to induce *Tbx3* and *Nanog* transcript expression, is increased in Ecad^−/−^ mESCs, however, no changes in Akt phosphorylation were observed in mESCs treated with DECMA-1 or Epep (data not shown). Addition of inhibitors of the PI3K/AKT, TGFβ and MEK/ERK pathways to Epep- and DECMA-1-treated cells identified several pathways associated with transcript expression/repression. In Epep-treated cells, *Klf4* transcripts are repressed via the TGFβ pathway, *Esrrb* transcripts are repressed via the PI3K/AKT pathway and Nr5a2 repressed via the TGFβ and PI3K/AKT pathways. In DECMA-1 treated cells, inhibition of *Esrrb* transcripts could be induced by abrogation of the MEK/ERK pathway and *Tbx3* transcripts inhibited by abrogation of the PI3K/AKT pathway. Therefore, alterations in signalling pathways upon loss of E-cadherin mediated cell-cell contact in mESCs are dependent upon the type of E-cadherin inhibitor employed. To date, only regions of E-cadherin that facilitate cell-cell contact, or compounds that can induce this loss, have been investigated in detail and it is possible that other regions of E-cadherin may also function to regulate signalling pathways in mESCs. Elucidating the function of these pathways alterations may provide critical insight into the role of E-cadherin function in stem cell and epithelial cell biology.

We have previously shown that pluripotency is maintained in Ecad^−/−^ mESCs and ENPS cells (mESCs exhibiting *de novo* methylation of the E-cadherin promoter) through the TGFβ signalling pathway^14^,^24^, specifically via Activin/Nodal, and that self-renewal is regulated via the FGFR1 pathway. However, mESCs treated with Epep or DECMA-1 did not induce Activin/Nodal-dependent pluripotency or FGFR1-dependent regulation of self-renewal despite ablation of STAT3 phosphorylation with both E-cadherin inhibitor treatments. Guo *et al*.^19^ have observed that disruption of E-cadherin mediated cell-cell contact does not influence many intracellular protein interactions with E-cadherin and concluded that most of these interactions are independent of cell-cell adhesion^19^. Therefore, it is possible that Epep and DECMA-1 inhibition of E-cadherin in mESCs does not disrupt cytoplasmic E-cadherin-protein interactions sufficiently for the switch to Activin/Nodal-dependent pluripotency and FGFR1-dependent self-renewal observed in Ecad−/− mESCs and ENPS cells^14^,^24^. Alternatively, this may reflect the activation of unknown signalling pathways capable of maintaining pluripotency and self-renewal in these cells.

Treatment of mESCs with Epep, Epep∆ctm or DECMA-1 resulted in increased viable cells within the population compared to control- and EpepW2R-treated cells. The AnnexinV/PI staining protocol requires a single cell suspension to perform the analysis, therefore, these results reflect the viability of single cells in suspension rather than in monolayer culture conditions. For example, no significant difference in cell viability was observed in mESCs treated with Epep or EpepW2R in monolayer culture (data not shown). Therefore, inhibition of E-cadherin in mESCs enhances single cell survival in suspension. We have previously shown that mESCs can be successfully cultured as near-single cell suspensions in shake flask culture using DECMA-1 treatment, demonstrating the usefulness of E-cadherin inhibition in bioreactor culture methods. However, the mechanisms for increased mESC viability in suspension appear to be distinct for Epep, Epep∆ctm and DECMA-1 treatments, again suggesting that specific regions of E-cadherin exhibit discrete cell survival functions in mESCs.

We show that abrogation of cell-cell contact by Epep was reversible and that *Nanog, Esrrb, Tbx3* and *Tet1* transcript expression was restored to control levels 24 h post-removal of the peptide inhibitor treatment. However, *Klf4, Nr0b1* and *Nr5a2* transcripts remained inhibited following removal of the peptide and this was also observed in DECMA-1 treated mESCs. Our previous analysis of Ecad−/− mESCs has demonstrated over 3000 transcripts alterations compared to wtD3 mESCs, showing that significant changes in transcriptional regulation and, presumably, epigenetic alterations are induced following loss of E-cadherin in these cells. The failure of *Klf4, Nr0b1* and *Nr5a2* transcripts to be restored to control levels following removal of the E-cadherin inhibitors suggests non-reversible epigenetic alterations may be occurring following inhibition of this protein. It would be interesting to assess the effect of multiple treatment and removal regimens of E-cadherin inhibitors to determine any additive effects on the transcriptome of mESCs, and other cell types. As such, the observation of potential non-reversible epigenetic effects upon E-cadherin inhibition may have relevance to inducing novel mESC lines exhibiting distinct transcriptomes and in the function of E-cadherin protein in tumorigenesis and cancer cell metastasis.

## Materials and Methods

### mESC culture and peptide treatment

Unless otherwise stated, mESC lines were grown on gelatinised tissue culture grade 6-well plates at 37 °C/5% CO_2_, as previously described^24^. mESCs cells were treated with 10 μM peptide in mESC culture medium which was replenished every 24 h. Inhibition of specific pathways in mESCs was achieved by incubation with 10 μM LY294002 (PI3K inhibitor), PD9805 (MEK inhibitor) and SB431542 (TGFβ1 R inhibitor).

### Peptide synthesis

All peptides were synthesised by Severn Biotech Ltd (UK) as a lyophilized powder. Peptides were solubilised to a stock solution of 100 mM in ddH_2_0, unless otherwise stated. Peptides were tagged with a Fluorescin-5(6) molecule at the NH_2_-terminus, separated by a Nova linker molecule, and assessed for activity prior to use (inhibition of cell-cell contact).

### Fluorescence flow cytometry analysis

D3 and Ecad^−/−^ mESCs were treated with Epep-F or EpepW2R-F at concentrations of 0 (control), 5, 10, 25, 50 and 500 μM in culture medium as a monolayer on ice. Ice cold conditions were utilised to minimise endocytosis of fluorescent tagged peptide. Cells were harvested using cell dissociation buffer, centrifuged and washed with PBS and fixed in 500 μl 1% w/v paraformaldehyde (PFA) in PBS for analysis using a FACSCalibur (BD Biosciences, California, USA). Viable cells were gated using forward scatter and side scatter and all data shown represents quantification of the fluorescence emitted by this population.

### Annexin V/PI assays

Cell viability was quantified using an Alexa Fluor 488 Annexin V/Dead Cell Apoptosis Kit (Invitrogen) according to the manufacturer’s instructions.

### Quantitative (q)PCR analysis

Primers for qPCR were designed using the Roche Applied science Universal Probe Library software and BLAST screened for sequence specificity. Only primers producing a single dissociation peak were used. RNA was extracted from mESCs and cDNA at a concentration of 2 ng/μl was used for analysis in a MicroAmp Optical 96-well Reaction Plate (Applied Biosystems) using SYBR green Jumpstart Taq ReadyMix (Applied Biosystems). Primer sequences: 18srRNA Forward (**F**) GTAACCCGTTGAACCCCATT, Reverse (**R**) CCATCCAATCGGTAGTAGCG; ESRRB **F-**GGCGTTCTTCAAGAGAACCA, **R**-ATCTGGTCCCCAAGTGTCAG; KLF4 **F-**GCTCCTCTACAGCCGAGAATC, **R**-ATGTCCGCCAGGTTGAAG; NANOG **F-**CACCCACCCATGCTAGTCTT, **R**-ACCCTCAAACTCCTGGTCCT; NR0B1 **F**-TGCACTTCGAGATGATGGAG, **R**-ATCTGCTGGGTTCTCCACTG; NR5A2 **F**-AGATGCCAGAAAACATGCAA, **R**-TGAGACATGGCTTCCAGCTT; TBX3 **F**-AGGAGCGTGTCTGTCAGGTT, **R**-GCCATTACCTCCCCAATTTT; TET1 **F**-GTTACGGAGAAGCGTGAAGC, **R**-TGCAGGTACGCTTTTTGTTG.

### Western blot analysis

Cells were harvested using cell dissociation buffer, centrifuged and washed with PBS prior to lysis using Radioimmunoprecipitation (RIPA) buffer (Sigma) containing protease inhibitor cocktail tablets (1 tablet per 10 ml RIPA buffer) (Roche Applied Science) at a concentration of 2 × 10^7^ cell/ml. Lysates were separated by SDS-PAGE and electrotransferred onto nitrocellulose membrane as previously described^24^. The membrane was probed using the appropriate primary antibody with subsequent incubation with HRP-conjugated secondary antibody. The membrane was exposed to an autoradiographic film (Hyperfilm ECL, GE Healthcare, Amersham, UK) for the appropriate time period in an X-ray cassette before the film was manually developed in a dark room using Kodak GBX developer and fixer solutions (Sigma, Dorset, UK). Primary antibodies: anti-α-tubulin, 1:2000 dilution (T9026, Sigma, Dorset, UK); anti-AKT, 1:200 dilution (#9272, Cell Signalling Technology, MA, USA); anti-pAKT (Ser473), 1:200 (#4060S, Cell Signalling Technology, MA, USA); anti-STAT3, 1:200 dilution (SC-482, Santa Cruz Biotechnology Inc, TX, USA); anti-pSTAT3, 1:200 dilution (#9138S, Cell Signalling Technology, MA, USA).

### Immunofluorescence microscopy analysis

Monolayer cultured mESCs were fixed *in situ* in 4% w/v PFA in PBS for 30 minutes at room temperature, washed twice with PBS and blocked with 1% goat serum in 0.1% w/v BSA, 0.1% Triton X-100 in PBS for 30 minutes. Cells were incubated in the appropriate primary antibody in goat blocking buffer for two hours at RT. Cells were washed three times in PBS and incubated in the appropriate Alexa fluor-488 or −546 conjugated secondary antibody at RT for 1 hour in the dark. Cells were washed three times with PBS, mounted in 4′6-diamidino-2-phenylindole (DAPI) (Vector Laboratories) and visualized using a Leica DM6000B fluorescence microscope (Leica Microsystems) and processed using Leica LAS X Core software (Leica Microsystems). Primary antibodies: anti-Nanog, 1:200 dilution (ab80892, Abcam Plc, Cambridge, UK); anti-Oct4, 1:200 dilution (ab19857, Abcam Plc, Cambridge, UK). For immunofluorescence microscopy analysis of fluorescent-tagged peptides, cells were incubated in 500 μM peptide in cell culture medium for 15 minutes, washed twice in ice cold PBS and mounted with Vectorshield containing DAPI. Cells were visualized using a Leica DM6000B fluorescence microscope (Leica Microsystems) and processed using Leica LAS X Core software (Leica Microsystems).

### Fluorescence analysis using ImageJ

Images were imported into ImageJ and fluorescence intensity measured using the Analyse > Measure function on areas selected using the rectangular or oval selection icons.

### Biacore Surface Plasmon Resonance (SPR) analysis

Biacore SPR analysis was used to measure the interaction between peptides and recombinant mouse E-Cadherin Fc Chimera (R&D Systems). Analytes were passed over an HTE chip with surface bound His-tagged Recombinant Mouse E-Cadherin Fc Chimera (R&D Systems). The kinetics of interaction, *i.e*. the rates of complex formation (ka) and dissociation (kd), were determined and Integrin α4β7 protein was used as a control. Analysis was performed by the Biomolecular Analysis Core Facility (The University of Manchester, UK) on a Proteon XPR-36 protein interaction array system (Bio-Rad) using a HTE chip for high capacity His-tagged protein capture (Bio-Rad). Equilibrium analysis runs were performed three times and an average KD was calculated. Running buffer: HBS/0.02%, Tween/2 mM, CaCl_2_/1 mM MgCl_2_.

## Additional Information

**How to cite this article**: Segal, J. and Ward, C. M. Novel peptides for deciphering structural and signalling functions of E-cadherin in mouse embryonic stem cells. *Sci. Rep.* **7**, 41827; doi: 10.1038/srep41827 (2017).

**Publisher's note:** Springer Nature remains neutral with regard to jurisdictional claims in published maps and institutional affiliations.

## Supplementary Material

Supplementary Figures Text

Supplementary Data
