# Supplementary material for: Novel peptides for deciphering structural and signalling functions of E-cadherin in mouse embryonic stem cells
Source: Sci Rep. 2017 Feb 7;7:41827. doi: 10.1038/srep41827 (PMC5294416; doi:10.1038/srep41827)
Supplement: Supplementary Figures Text [file srep41827-s1.doc]

**Novel peptides for deciphering structural and signalling functions of**

**E-cadherin in mouse embryonic stem cells**

Joe Segal and Christopher M Ward

**Supplementary text**

**Supplementary Figure S1.** (a) qPCR analysis of *Nanog*, *Klf4*, *Tbx3*, *Esrrb*, *Nr0b1* and *Nr5a2* transcript expression in mESCs treated with Epep and DECMA-1 and cultured in the presence of small molecule inhibitors of the TGFβ, MEK and AKT pathways. Green arrows show upregulated transcripts and red arrows show downregulated transcripts. (b) ImageJ analysis of loss of cell-cell contact in mESCs treated with control vehicle (wtD3), Epep or EpepP7R. Light intensity between cells was measured in phase contrast images of the treated mESCs in ImageJ. Data shows the mean light intensity and standard deviation. (c) ImageJ analysis of loss of cell-cell contact in mESCs treated with control vehicle (wtD3), Epep, EpepS1A, EpepW2A, EpepE3A, EpepL4A, EpepY5A or EpepY6A. Light intensity between cells was measured in phase contrast images of the treated mESCs in ImageJ. Data shows the mean light intensity and standard deviation. (d) Quantification of NANOG protein expression in the images shown in Figure 6c using ImageJ analysis. Data shows the mean fluorescence intensity and standard deviation.

**Supplementary Figure S2.** ImageJ quantification of immunofluorescence microscopy analysis of fluorescently labelled EpepS1A, EpepW2A, EpepE3A, EpepL4A, EpepY5A and EpepY6A binding to wtD3 mESCs and Ecad^-/-^ mESCs following incubation at 500µM for 15 minutes at 37˚C. Data shows the mean fluorescence intensity and standard deviation.
