# Supplementary figures and images for: Novel peptides for deciphering structural and signalling functions of E-cadherin in mouse embryonic stem cells
Source: Sci Rep. 2017 Feb 7;7:41827. doi: 10.1038/srep41827 (PMC5294416; doi:10.1038/srep41827)

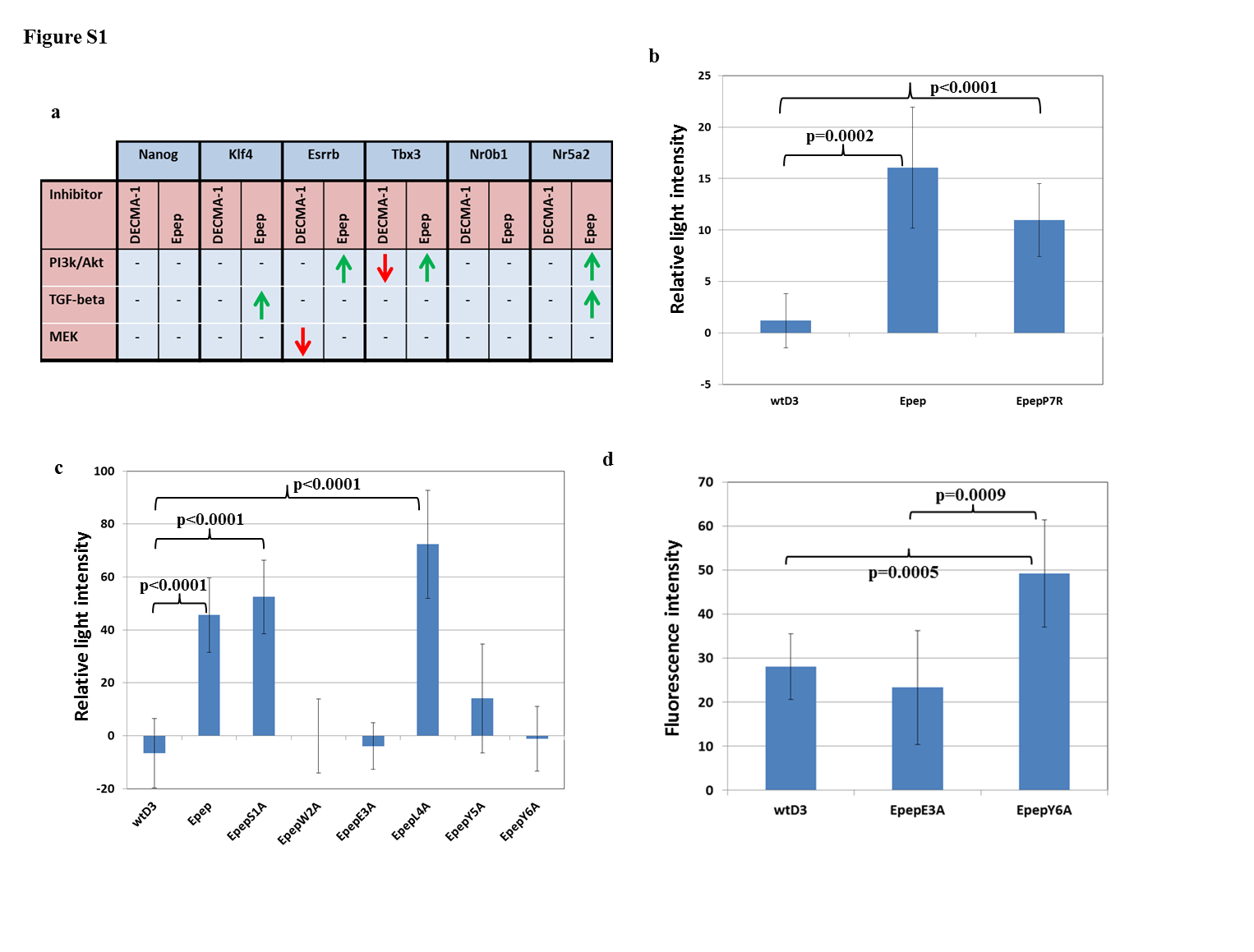


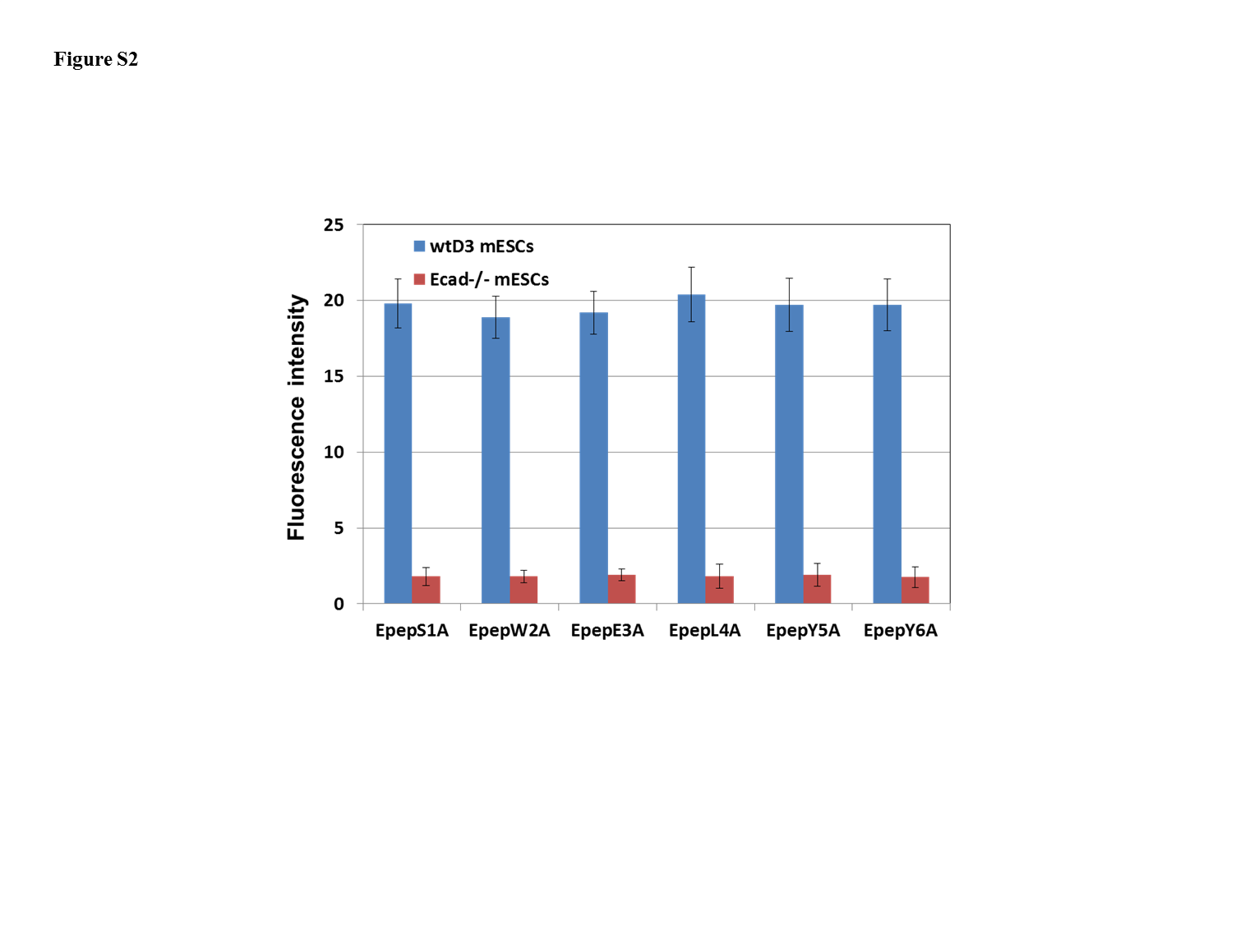

Supplement: Supplementary Data [file srep41827-s2.doc]
